# New Insights into Lymphocystis Disease Virus Genome Diversity

**DOI:** 10.3390/v14122741

**Published:** 2022-12-08

**Authors:** Jessica Benkaroun, Sven M. Bergmann, Angela Römer-Oberdörfer, Menekse Didem Demircan, Cüneyt Tamer, Gayatri Rajendra Kachh, Manfred Weidmann

**Affiliations:** 1 Institute of Aquaculture, University of Stirling, Stirling FK9 4LA, Scotland, UK; 2 Institute of Infectology, Friedrich-Loffler-Institute (FLI), Federal Research Institute for Animal Health, 17493 Greifswald, Germany; 3 Jockey Club College of Veterinary Medicine, City University of Hong Kong, Hong Kong; 4 Faculty of Aquatic Sciences, Istanbul University, 34130 Laleli/İstanbul, Turkey; 5 Department of Virology, Faculty of Veterinary Medicine, Ondokuz Mayis University, 55200 Samsun, Turkey; 6 Medizinische Hochschule Brandenburg Theodor Fontane, 01968 Senftenberg, Germany

**Keywords:** lymphocystis disease virus, LCDV1, whole genome, nucleocytoplasmic large DNA virus, demersal fish

## Abstract

Lymphocystis disease viruses (LCDVs) are viruses that infect bony fish which has been found in different locations across the globe. Four virus species have been classified by the International Committee on Taxonomy of Viruses (ICTV), despite remarkable discrepancies in genome size. Whole genome sequencing and phylogenetic analysis of LCDVs from wild fish from the North Sea and partial sequences from gilthead sea bream of an aquafarm located in the Aegean Sea in Turkey confirm that the LCDV1 genome at 100 kb is approximately half the size of the genomes of LCDV2-4. Since the fish species, of which LCDV1 was isolated, differ taxonomically at the order level, co-speciation can be excluded as the driver of the adaptation of the genome of this nucleocytoplasmic large DNA virus, but may represent an adaptation to the lifestyle of this demersal fish in the northeast Atlantic.

## 1. Introduction

Lymphocystis disease viruses (LCDVs) are a member of the *Iridoviridae* family, which comprises two subfamilies, *Alpha-* and *Betairidovirinae*. Viruses of the genera *Lymphocystivirus* and *Megalocytivirus* infect fish [1,2].

Described since the 19th century, the viral cause of the disease in fishes was suggested in 1914, and LCDV was first isolated in 1962 [3,4]. More than 140 wild fresh and marine water fish, amphibians, and reptiles, as well as ornamental fish, have been reported to be affected worldwide. The virus prevalence of LCDV can be very high, especially in aquaculture systems [2].

In recent years, mortalities have occurred in farmed gilthead sea bream (*Sparus aurata*) [5,6,7] in the Mediterranean Sea, and in olive flounder (*Paralichthys olivaceus*) [8,9] and Japanese seabass (*Lateolabrax japonicus*) in waters off the coasts of Japan and Korea [10,11]. In the Mediterranean Sea, LCDV was also isolated from farmed Senegalese sole (*Solea senegalensis*) [5].

In fish farming conditions, the virus is known to cause lymphocystis diseases, which results in skin outgrowths (nodes) covering the whole fish body. The virus itself is not associated with high mortality, but opportunistic infections can occur following LCDV infection and compromise overall fish health.

According to the ICTV taxonomy of viruses, the Lymphocystisvirus genus comprises four virus species: LCDV1 was isolated from the European flounder (*Platichthys flesus* (108 Kbp)), LCDV2 was isolated from *Paralichthys olivaceus* (186 Kbp), LCDV3 was isolated from *Sparus aurata* (208 Kbp), and LCDV4 was isolated from *Micropogonias furnieri* (211 kb) (Table 1, [3]).

The genome of the different virus species varies considerably in size. LCDV1 has been characterised with a genome of ~100 Kpb in size while LCDV2–4 have a genome double this size. Their double-stranded DNA genome contains terminal repeated regions and is highly methylated, which seems to be a unique feature of this genus. The four virus species have been grouped as a genus by the ICTV based on the common presence of a gene subset, a certain percentage of nucleotide and amino acid identities, and the level of genomic methylation, but not on the overall genomic organisation.

Here we report the whole genome characterisation by sequencing and the comparative analysis of complete genomes from LCDV isolates obtained during a virus surveillance conducted on wild fish captured in the North Sea in 2003 and of a LCDV genome from a PCR-positive gilthead sea bream collected from a LCDV-positive aquafarm in the Aegean Sea, in Turkey.

The current LCDV classification is based on the whole genomic structure and organisation of the virus, host range, nucleotide identities, and gene/protein annotations. We provide new evidence which suggests that LCDV1 may be a separate virus from LCDV2–4.

## 2. Materials and Methods

### 2.1. Virus Isolation and Fish Samples

The Friedrich-Loeffler-Institut (FLI) team caught wild fish in the North Sea between 1996 to 2000. Tissue samples from wild fish (the common dab (*Limanda limanda*), European plaice (*Pleuronectes platessa*), European flounder (*Platichthys flesus*), Long rough dab (*Hippoglossoides platessoides*), and gray gurnard (*Eutrigla gurnardus*) showing severe clinical signs of lymphocystis disease (LCD) were tested using PCR. Additionally, LCDV was isolated in fish cell line SAF-1 [9] from the common dab, European plaice, European flounder, and gilthead sea bream (*Sparus aurata*) but the isolates were lost due to a freezer breakdown. The Faculty of Aquatic Sciences at Istanbul University provided ten deceased gilthead sea bream (5–25 g) with conspicuous nodes on their skin collected in February 2012–2016 at adaptation facilities and sea cages in the Aegean Sea with confirmed LCDV outbreaks. Tissues PCR tests were performed as described below; virus isolation attempts on SAF-1 cells [9] failed.

### 2.2. LCDV DNA Extraction from Fish Tissues

Tissues from several samples of LCD diseased fish were ground using sterile sea sand and sterile isotonic buffer. This suspension was used for DNA extraction using the Trizol^®^ reagent (Invitrogen, Darmstad, Germany) by Qiamp DNAMini Kit (Qiagen, Hilden, Germany) (FLI) and using the Qiagen Dneasy Blood & Tissue Kit (Qiagen, Hilden, Germany) (Stirling), according to the manufacturer’s instructions. The extracted DNA was dissolved in 50 µL PCR-grade water and stored at −20 °C until their use. The DNA concentration was determined using the Nanodrop spectrometer and diluted to 10 ng/µL.

### 2.3. LCDV Detection

At the FLI, the PCR amplification of the MCP gene fragment was performed using the primers OBL 3 and OBL 4 (PCR) and OBL 5 and OBL 6 (nested PCR) gene as described [10].

At Istanbul University, PCR amplification of the MCP gene fragment was performed using 10 µM of published primers LCDVs-F, LCDVs-R [11], and 10 ng DNA sample in a Mytaq PCR mix, according to manufacturer’s recommendations (Bioline, London, UK), using the temperature profile 95 °C/5 min and 35 cycles of 95 °C/1 min, 55 °C/1 min, and 72 °C/1 min, with a final extension at 5 min/72 °C on a Biometra thermal cycler. The amplicons were analysed on an agarose gel.

### 2.4. Library Preparation and Illumina Sequencing

Libraries were prepared with the Illumina Nextera XT DNA Library Preparation Kit (Illumina, Cambridge, UK). The tagmentation reaction used 0.5 ng of each LCDV amplicon. The quality and quantification of each library were assessed using the High Sensitivity DNA chip and reagents (Agilent, Stockport, UK). The libraries were normalised at equimolar concentration and pooled manually. Paired-end sequencing was performed with a read length of 2 × 75 bp using the MiSeq Reagent Kit v3, with 150 cycles on the MiSeq sequencer (Illumina, Cambridge, UK).

### 2.5. Data Cleaning

The quality of raw sequencing reads were checked with the FastQC tool [12]. Illumina adapter sequences were clipped, and low quality reads were filtered and removed with Trimmomatic v.0.39 [13]. Over-represented sequences were removed with Cutadapt v3.1 [14]. Filtered reads were de novo assembled using SPAdes v.3.15.0 [15]. The resulting contigs were blast against the nucleotide database from NCBI [16].

### 2.6. Phylogenetic Analysis

The LCDV major capsid protein nucleotide sequences were retrieved using BLAST on NCBI [16]. All LCDV nucleotide sequences were aligned with MUSCLE, implemented in MEGA7 [17]. The phylogenetic tree of MCP gene sequences was inferred in MEGA with the neighbour-joining method with 1000 bootstrap replications. Evolutionary distances were computed using the maximum composite likelihood method and were represented by the number of base substitutions per site, scale bar. The evolutionary trees were visualised in FigTree [18]. The whole genome alignment was performed using MAFFT [19], and the bootstrapped phylogenetic tree of whole genome sequences was inferred using RAxML [20], both of which were included in the MEGALIGN module of the DNASTAR software package.

### 2.7. Whole Genome Comparative Analysis

The LCDV complete genomes were aligned using the Mauve Aligner using the default settings [21]. Homologous genomic regions are shown as coloured blocks. The Circular Genome Viewer was used to visualise the circular LCDV1 genomes [22]. A pairwise matrix of genomic distance was calculated using the Jukes–Cantor model.

## 3. Results

Samples obtained from the two dabs and one sample each from the flounder, long rough dab, plaice, and gray gurnard tested positive by MCP-nested PCR. The obtained MCP sequences showed a high homology to the LCDV1 with similarities close to 100% (Table 2).

**Figure 1:**
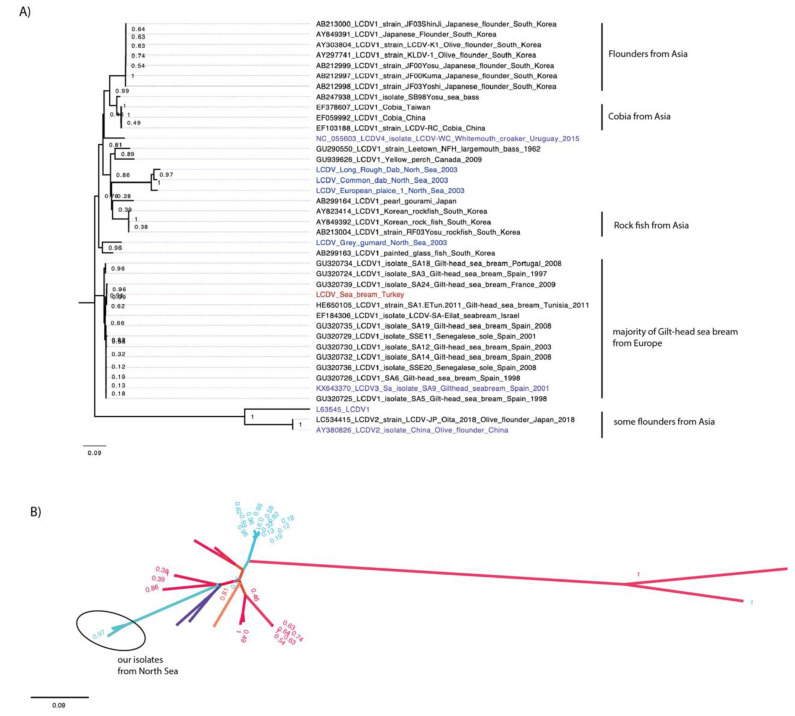
Phylogeny of the conserved MCP gene of LCDVs. Alignment of the new LCDV MCP gene sequences with other LCDV MCP gene sequences available from NCBI. Bootstrap values are indicated on tree branches. (**A**) LCDV1 to 4 are coloured in purple. MCP sequences from North Sea fish are coloured in blue and from Turkey in red. (**B**) Radial representation of the phylogenetic tree. Tree branches were coloured by locations. Viruses from Asia were colored in red, in blue from Europe, in orange from South America, and in purple from North America. The isolates of this study are encircled with a black line.

The initial analysis of the conserved major capsid protein gene (MCP) is traditionally used for Iridovirus phylogenetic analysis from partial genome sequences. The MCP sequence of the virus genomes we sequenced were analysed using BLAST against all MCP sequences from NCBI. Similar sequences identified from the BLAST were retrieved and aligned, and their relationships was investigated by phylogenetic analysis (Figure 1).

The phylogenetic analysis of all available, as well as the new, MCP gene nucleotide sequences showed that most of the viruses retrieved and compared were grouped by similar hosts (Figure 1A). All MCP sequences of the farmed gilthead sea bream isolates from different locations in Europe were clustered together. Similarly, the same pattern was seen for the MCP sequences of isolates from wild cobia fish from Asia, from farmed olive flounders from Asia, and from farmed rock fish from South Korea.

The LCDV sequences characterised from wild fish from the North Sea grouped together. All of these flat fish display similar behaviours and habitats, and are commonly found in the North Sea.

The only exception was the LCDV sequence of the isolate from a grey gurnard from the North Sea, which grouped with the sequence of a LCDV isolate from *Chanda baculis*, a tropical ornamental fish species from South Korea with a high bootstrap value of 0.96.

The LCDV sequence determined from he gilthead sea bream from Turkey grouped with sequences of LCDV1 isolates obtained from farmed gilthead sea bream elsewhere in the Mediterranean Sea. The radial representation of the MCP sequence phylogenetic tree indicated virus speciation, possibly due to different geographic locations (Figure 1B). Most viruses from Europe (coloured in blue) form a distinct group separate from viruses from Asia (coloured in red). The MCP sequences of the isolates from the North Sea were grouped in a distinct separate branch.

To better understand the difference between LCDV1 and LCDV2–4, we next conducted a whole genome analysis of the four LCDV types. (Table 3).

Three full LCDV genomes were obtained: LCDV-CD, 102,221 bp, LCDV-LRD 102,349 bp, and LCDV-EP, 102,677 bp, (accession no. OP745010-12), with mean sequencing coverage ranging from 14–25. The sequence results of the Turkish isolate only allowed us to assemble partial sequences and a full genome was not obtained (dataset available upon request).

**Figure 2:**
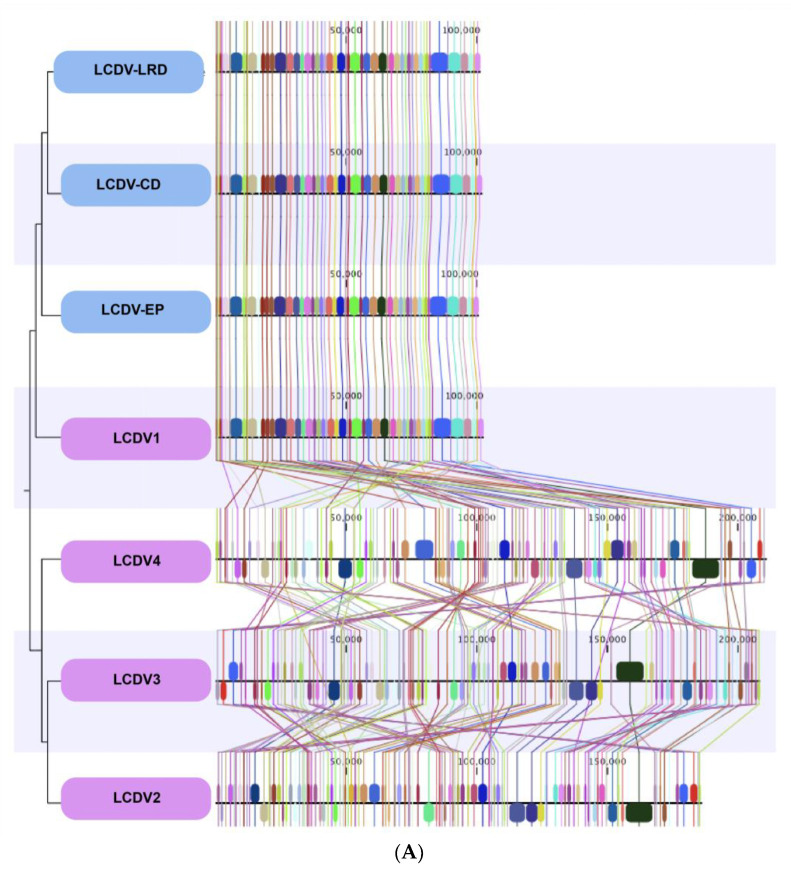

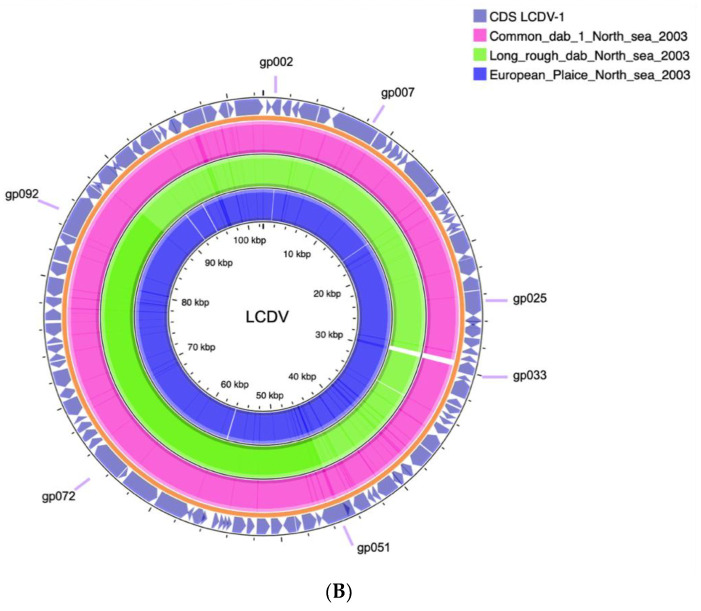
(**A**) Comparative MAUVE alignment of LCDV1, LCDV2, LCDV3 (LCDV-C), LCDV3, and LCDV4, and three LCDV complete genomes of LCDV-EP (European plaice), LCDV-LRD (Long rough dab), LCDV-CD (Common dab). Vertical coloured lines and blocks show the conservation of genomic regions across the genomic sequences. (**B**) Obtained LCDV genome sequences aligned to the virus species LCDV1 in a circular manner.

The complete published genomes of LCDV1-4 were retrieved and aligned with the three complete LCDV genomes sequenced from the isolates of wild fish caught in the North Sea (Figure 2).

The whole genomes of the LCDV isolates from the North Sea fish aligned closely with the previously described LCDV1 virus species genome with a very high nucleotide and genomic conservation compared to the other virus species LCDV2–4 (Figure 2A,B, Table 3).

All LCDV complete genomes that we sequenced displayed a similar genome size of ~100 Kbp and similar conserved genomic regions throughout their genomes with LCDV1, while the percentage of nucleotide identities in comparison to the virus species LCDV2–LCDV4 was close to zero, meaning almost complete genomic dissimilarities and a non-relatedness of the virus species. Some LCDV1 genomic regions were found in LCDV2-4 but at different locations within their genomes.

**Figure 3:**
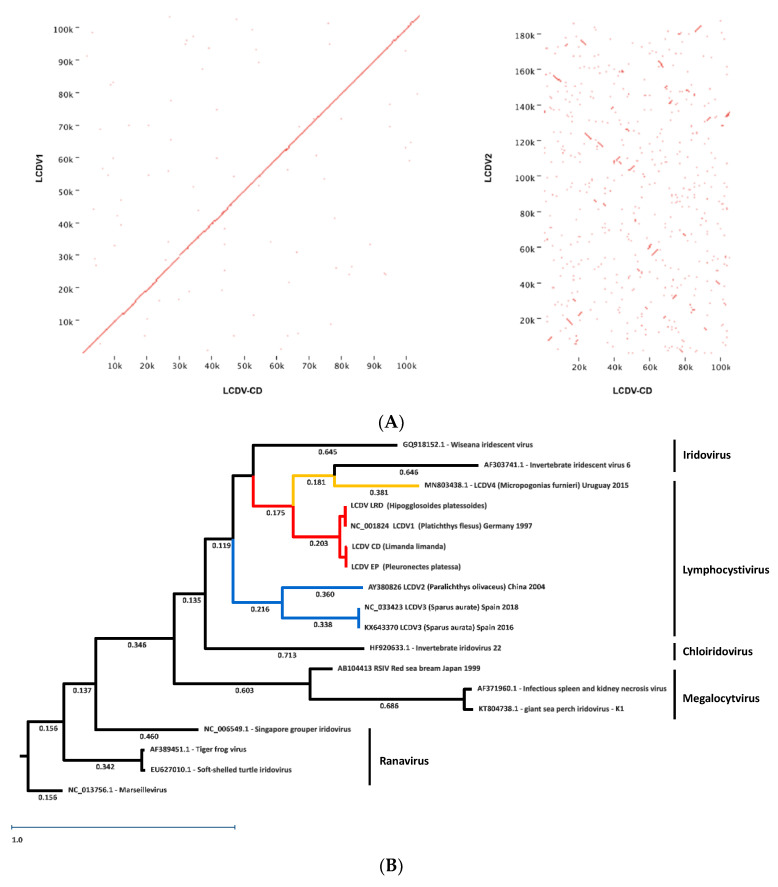
(**A**) Genomic dot plot of LCDV genomes. The left figure shows the comparison of the genomic sequences of LCDV-CD with LCDV1, the right figure shows the comparison of LCDV-CD with LCDV2. (**B**) RAxML maximum likelihood tree (1000 bootstraps) of full genome *Iridoviridae* sequences. Numbers depict posterior probability values. The bootstrap values are given in Figure S2. Choice of genomes for *Betairidoviridae* genera *Iridovirus* and *Chlroiridovirus* and *Alphairidoviridae* genera *Ranavirus* and non-*Iridoviridae* outgroup nucleocytoplasmic large DNA viruses Marseillevirus, according to [23]. LCDV1-4 are highlighted in red and orange; LCDV2-3 in blue.

An additional genomic dot plot analysis of the North Sea isolates LCDV-CD and LCDV1 yielded almost an identical alignment with a clear diagonal line at the centre of the plot, whereas an alignment with LCDV2 showed a complete dissimilarity and a non-diagonal line (Figure 3A).

A MAFFT alignment and RAxML tree of all LCDV and selected representative Iridovirus full genome sequences was inferred. It clearly shows a distinct clustering of the LCDV1 sequences from the LCD2–3 sequences but groups in a subclade with LCDV4.

A pairwise comparison of shared coding sequences (CDS) revealed that LCDV1 shares only three and four genes with LCDV2 and LCDV3, respectively, but 13 with LCDV4. The genome pair LCDV2–3 shares 24, while LCD3-4 shares 22 CDSs (Figure S1).

## 4. Discussion

The first LCDV genome described was isolated from *Platichthys flesus* (European flounder) a demersal flatfish found on the European shelf of the northeast Atlantic. [4,24]. In aquaculture, the virus research isolation and characterisation of LCDV2 and LCDV3 was driven by the impact that these viruses had in the culture of the demersal species *Sparus aurata, Dicentrarchus labrax*, and *Solea senegalensis* in European waters, and *Paralichthys olivaceus* and *Lateolabrax japonicus* in East-Asian waters.

Here, we describe the first partial LCDV3 genome sequence derived from tissue extract of gilthead sea bream from a marine aquafarm in Turkey. Given that gilthead sea bream is farmed in similar settings throughout the Mediterranean, it is not surprising that LCDV3 was also found in this sample. It is well-known that LCDV can occur asymptomatically in 30–100% of gilthead sea bream populations [25].

The study of the genomes of LCDV isolates from demersal fish of the North Sea, clearly shows that they are related only to LCDV1 with a high percentage of nucleotide identities (91–98%). The genome sequences of all LCDV1 isolates are clearly distinct from those of LCDV2–4, and nucleotide identities between the two groups range from only 30–39%. This was confirmed by dot plot analysis (Figure 3A). Additionally, a phylogenetic analysis of full *Iridoviridae* genomes indicated a distinct grouping of LCDV1/4 from LCDV2–3.

This is underpinned by the pairwise comparison of shared coding sequences (CDS) plotted in a Venn diagram, which revealed that LCDV1 shares few genes with any of LCDV2–4 and has the highest sharing rate with LCDV4 (Figure S1).

In the LCDV1/4 subclade, the 211 k sequence of LCDV4 pairs with the 212 k genome of Iridovirus IIV6. This can be attributed to a limited resolution in the current topology of the tree, which is supported by strong bootstrap values (Figure S2). The availability of more genomes in the future should allow a finer grained distinction between Iridovirus and Lymphcystisvirus.

The fish species from which LCDV1 has been isolated differ taxonomically at the order level (*Perciformes* and *Pleuronectiformes* [26]), and this may explain why previous attempts to relate the genetic diversity of LCDV to co-speciation with their hosts failed [27]. The first description of LCDV4 compared the amino acids sequences of the proteins of a subset of 26 concatenated core genes of all four LCDV types by pairwise sequence identity analysis, and LCDV1 yielded a sequence identity below 85% throughout [8]. This assessment can be extended to highly homologous LCDV1 sequences described here. Traditionally, this could already be deemed sufficient to define a new virus species within the genus *Lymphocystivirus* or even a new genus in *Alphairidovirinae.*

However, since the *Iridoviridae* belong to the nucleocytoplasmic large DNA viruses (NCLDV), an approach to classify by agreed sets of core genes has become common to be able to handle this complicated group of diverse viruses.

Constant reassessment of new NCLVD virus sequences indicates that these viruses have possibly evolved by accumulating genes from their hosts through lateral gene transfer events. NCLDV genome ORFs are either unique, species-specific, or form unique protein clusters within the NCLDV virus. Evidence of gene sharing in vertebrate-infecting NCLDVs is confined to *Iridoviridae*- and *Poxvididae*-infecting fish (and amphibians). The share of unique protein clusters does not correlate with genome size or ORF number, and the virus genome size seems to enlarge or shrink depending on environmental factors through insertions, deletions, and duplications [28]. It is possible that the differences in the LCDV1 genomes, as opposed to the LDV2-4 genomes, might be due to these mechanisms of rearrangement.

It is well-documented that unlike terrestrial viruses, viruses infecting fish are very promiscuous in their choice of host and are usually able to infect a wide array of fish species [29,30,31]. Strikingly, all described LCDV1–4 genomes have been isolated from demersal fish, indicating that it is not their taxonomy that ranges across several orders, but their lifestyle which is the common denominator (Table 4).

It is therefore tempting to conclude that unlike the double-sized genome of LCDV2–3, isolated from demersal fish of the Mediterranean and the western Pacific on the one hand, and of LCDV4 from the Atlantic board of South America on the other hand, the LCDV1 genome might be an adaptation of the nucleocytoplasmic large DNA virus LCDV to the demersal life style of their northeast Atlantic hosts on the European shelf, as indicated by LCDV1 and 4 grouping in the same clade (Figure 3A).

The evidence, in regards to homology and encoded ORFs, indicate a distinct LCDV1 genome as compared to LCDV2-4. The only common denominator among all LCDV genomes is the CDS for DNA methyltransferase. This CDS and the level of genomic methylation, not the genome structure, had been a characteristic originally chosen by ICTV to define this group. Since a recent analysis yielded no predictable methylation sites in LCDV1 and LCDV2-3 genome sequences, even this characteristic feature appears unsustainable [23].

The argument of connecting the observed distinct genome structure of LCDV1, with the lifestyle of its diverse hosts, should justify to create a new genus in the *Alphairidovirinae* for the 100 kb-sized LCDV1 virus replicated in demersal fish. To conclude, we suggest that LCDV1 should be classified as a separate virus from LCDV2–4 due to strong discrepancies in its genomic size and organisation.

## Figures and Tables

**Table 1:** Current LCDV complete genomes publicly available.

Virus Name (Isolate)	Genome Size (bp)	Accession- No.	Country (Date)	Reference	Host
LCDV1	102,653	NC_001824	Germany (1983)	[4]	*Platichthys flesus*
LCDV2 (LCDV-JP)	186,627	LC534415	Japan (2018)	[5]	*Paralichthys olivaceus*
LCDV2 (LCDV-C)	186,250	NC_005902	China (2000)	[6]	*Paralichthys olivaceus*
LCDV3 (LCDV-Sa9)	208,501	NC_033423	Spain (2001)	[7]	*Sparus aurata*
LCDV4 (LCDV-WC)	211,086	NC_055603	Uruguay (2015)	[8]	*Micropogonias furnieri*

**Table 2:** Comparison of sequence similarities of partial MCP sequences (231–263 bp) obtained using nested PCR from different wild fish species caught in the North Sea between 1996 and 2003. LCDV1 (NC_001824).

	Dab 1	Dab 2	Flounder	Long Rough Dab	Plaice	Gray Gurnard	LCDV1
Dab 1	-	100%	97%	99%	99%	100%	99%
Dab 2	100%	-	97%	99%	98%	99%	99%
Flounder	97%	97%	-	98%	98%	99%	98%
Long rough dab	99%	99%	99%	-	98%	99%	99%
Plaice	98%	98%	98%	98%	-	99%	99%
Gray gurnard	99%	99%	99%	100%	99%	-	99%
LCDV1	99%	99%	98%	99%	99%	99%	-

**Table 3:** LCDV pairwise genomic distance. The degree of genome relatedness ranges from warmer to cooler colors. Percentage identities are shown on the upper side of the table, and genetic distances are shown on the lower side. Genetic distances will range from 0 to 1: 1 represents identical genomic sequences; 0 means that genomic sequences are very dissimilar.

	LCDV1	LCDV-CD	CDV-LRD	LCDV-EP	LCDV2	LCDV3	LCDV4
LCDV1	-	92.93	93.49	98.06	0	0	0
LCDV-CD	0.98	-	93.62	91.54	0	0	0
LCDV-LRD	0.97	0.98	-	91.75	0	0	0
LCDV-EP	0.98	0.97	0.97	-	0	0	0
LCDV2	0.39	0.39	0.32	0.31	-	82.28	83.6
LCDV3	0.30	0.30	0.30	0.30	0.24	-	82.67
LCDV4	0.30	0.30	0.0043	0.31	0.39	0.24	-

**Table 4:** LCDV genome types and their hosts.

Species	Order	Life Style *	Location	Aquaculture Isolates	LCDV Species
*Platichthys flesus* (European flounder)	* Pleuronectiformes *	Marine; freshwater; brackish; demersal; catadromous; 1–100 m	Northeast Atlantic	no	LCDV1
*Limanda limanda* (Common dab)	* Pleuronectiformes *	Marine; demersal; oceanodromous 20–150 m	Northeast Atlantic	no	LCDV1
*Pleuronectes platessa* (European plaice)	* Pleuronectiformes *	Marine; brackish; demersal; oceanodromous 10–50 m	Northeast Atlantic	no	LCDV1
*Eutrigla gurnardus* (Grey gurnard)	* Perciformes *	Marine; brackish; demersal; 10–340 m	Northeast Atlantic	no	LCDV1
*Hippoglossoides platessoides* (Long rough dab)	* Pleuronectiformes *	Marine; demersal; oceanodromous 10–3000 m	Northeast and northwest Atlantic	no	LCDV1
*Paralichthys olivaceus* (Olive flounder)	* Pleuronectiformes *	Marine; demersal; oceanodromous 10–200 m	Western Pacific	yes	LCDV2
*Lateolabrax japonicus* (Japanese seabass)	* Pempheriformes *	Marine; freshwater; brackish; reef-associated; catadromous; 5–? m	Western Pacific	yes	LCDV2
*Sebastes schlegelii* Korean rockfish	* Perciformes *	Marine; demersal; 3–100 m	Western Pacific	yes	LCDV2
*Sparus aurata* (Gilthead sea bream)	* Spariformes *	Marine; brackish; demersal; 1–150 m	Eastern Atlantic and Mediterranean	yes	LCDV3
*Solea senegalensis* (Senegalese sole)	* Pleuronectiformes *	Marine; demersal; depth range 12–65 m	Eastern Atlantic and Mediterranean	yes	LCDV3
*Micropogonias furnieri* (Whitemouth croaker)	* Blenniiformes *	Marine; brackish; demersal; oceanodromous; ?–60 m	Atlantic board of Central and Southern America.	no	LCDV4

* from https://www.fishbase.se accessed on 18 April 2022.

## Data Availability

Not applicable.

## Supplementary Materials

The following supporting information can be downloaded at: https://www.mdpi.com/article/10.3390/v14122741/s1, Figure S1 Venn diagram of shared coding sequences of LCDV 1-4.
