# Supplementary material for: New Insights into Lymphocystis Disease Virus Genome Diversity
Source: Viruses. 2022 Dec 8;14(12):2741. doi: 10.3390/v14122741 (PMC9781669; doi:10.3390/v14122741)
Supplement: Supplementary file 1 [file viruses-14-02741-s001.zip › viruses-1974030-Supplementary.pdf]

viruses

MDPI

Supplementary Information

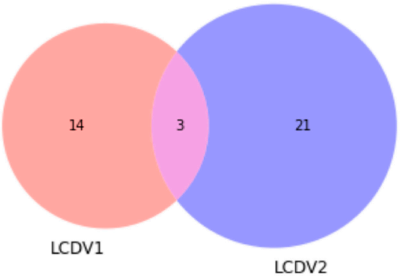

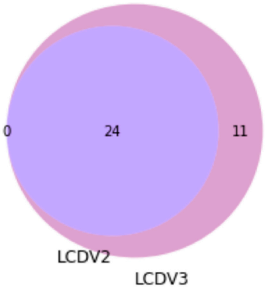

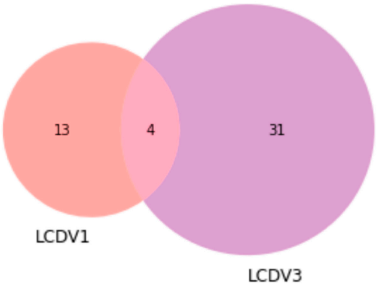

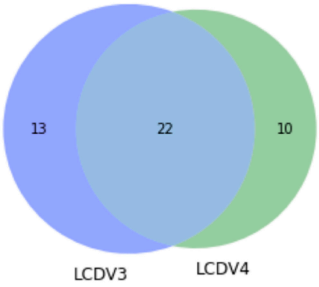

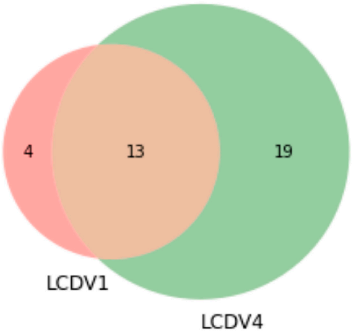

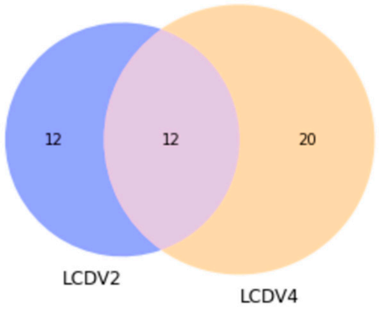

Figure S1
Venn diagram of shared coding sequences of LCDV 1-4. Venn diagrams were plotted using the matplotlib-venn 0.11.7 python library. LCDV1-4 ORFs were screened from overall 111, 239,366, 148 ORFs enoded by the respective LCDV1-4 genomes.

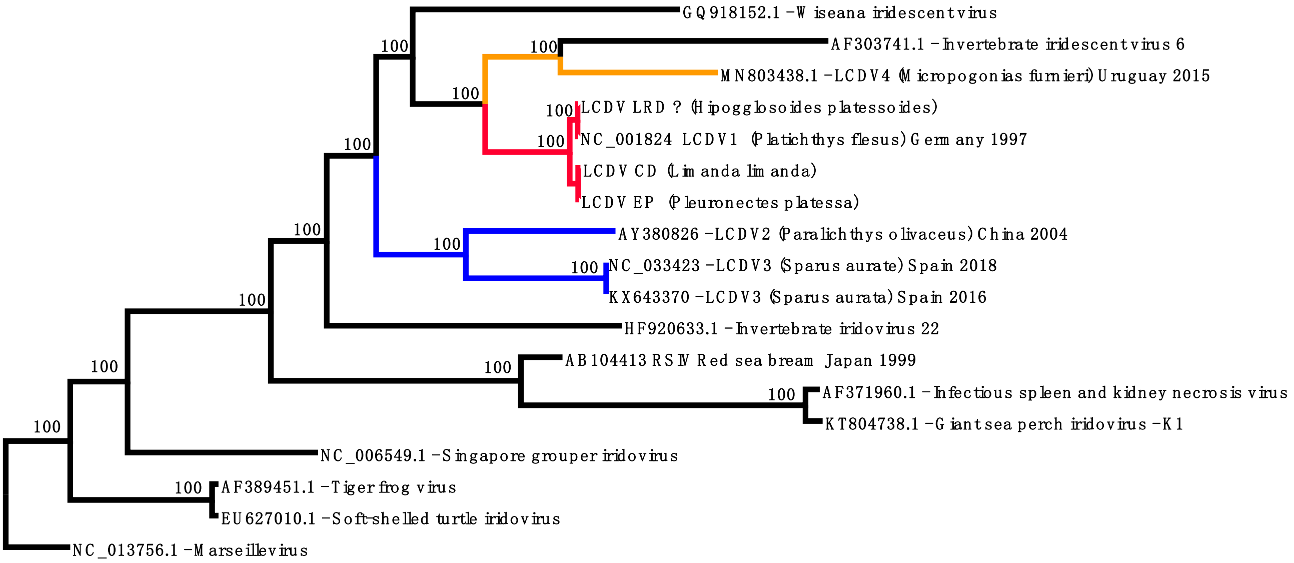

Figure S2: RaXML tree of Figure 3A presented with bootstrap values given in 100%.
